# Influence of deep learning-based journal reading guidance system on students’ national cognition and cultural acceptance

**DOI:** 10.3389/fpsyg.2022.950412

**Published:** 2022-08-25

**Authors:** Wei Huang, Fangbin Song, Shenyu Zhang, Tian Xia

**Affiliations:** ^1^School of Arts, Southeast University, Nanjing, China; ^2^School of Design Art and Media, Nanjing University of Science and Technology, Nanjing, China; ^3^School of Liberal Arts, Nanjing Normal University, Nanjing, China; ^4^School of Economics, Southwestern University of Finance and Economics, Chengdu, China

**Keywords:** educational psychology, art journal, national identity, guidance system, deep learning

## Abstract

The purpose is to explore new cultivation modes of college students’ national cognition and cultural acceptance. Deep learning (DL) technology and Educational Psychology theory are introduced, and the influence of art journal reading on college students’ national cognition and cultural acceptance is analyzed under Educational Psychology. Firstly, the background of Educational Psychology, national cognition and cultural acceptance, and learning system are discussed following a literature review. The DL technology is introduced to construct the journal reading guidance system. The system can provide users with art journals and record the user habits like reading duration and preferences. Secondly, hypotheses are proposed, and a questionnaire survey is designed, with 12 specific indicators to investigate and collect research data. Finally, the collected data are analyzed. The results show that women’s cognition of Chinese traditional culture, Chinese excellent revolutionary culture, and Chinese national identity is higher than that of men. By comparison, men’s cognition of Chinese advanced socialist culture is higher than women’s. After using the journal reading guidance system, the cognition of female college students on traditional Chinese culture is improved by 16.3%. Before and after reading art journals, the overall national cognition and cultural acceptance of Minority students are higher than that of Han students. The overall cognition of Literature and History students is higher than that of Science and Engineering students in traditional Chinese culture and China’s excellent revolutionary culture and lower in advanced Chinese socialist culture and Chinese national identity. The overall cognition of college students’ party members to the advanced socialist culture is higher than league members. As students read more art journals through the guidance system, their overall national cognition and cultural acceptance have increased. Therefore, reading art journals can promote college students’ national cognition and cultural acceptance. A national cognition and cultural acceptance promotion system that conforms to the current situation of college students is constructed. The finding provides a reference for developing complex emotion recognition technology in human-computer interaction.

## Introduction

### Research background and motivations

The 21st century marks improved spiritual wellbeing and higher demand for the comprehensive personality of humanity. As a result, more attention has been paid to quality education (Zhou et al., 2019). Studying Educational Psychology is of great significance (Graesser et al., 2022), given that difficult employment situations and social competition have caused psychological problems among college students (Paananen et al., 2021). Cultivating college students’ physical and mental health and helping them socialize have always been researched hotspots (McMaster et al., 2021). These concern the social and national interests. Common psychological problems among college students include personality defects and emotional disruption. More precisely, they are cowardice, paranoia, aggression, inferiority complex, and unwarranted worry (Carvajal et al., 2021). Studies have shown that Educational Psychology can well assist psychotherapy and prevent mental illness (Niu et al., 2020; Tummala-Narra et al., 2021). Traditionally, theoretical art education teaches students to shape correct values by appreciating art theory and history (Taliaferro et al., 2020). College students, as the new force of the country, should cultivate their aesthetic and innovation abilities. Reading art journals might shed light on college art education reform by improving their national cognition and cultural acceptance (Wright et al., 2021).

Chinese art journals are diverse. For example, journals like *Literature Art Studies* and *Hundred Schools in Arts* explore literature, drama, film and television, and plastic arts. *The decoration* is famous in the field of arts and crafts. Also, there are *Art Research*, *New Arts*, *Art Magazine*, and *Journal of Nanjing Arts Institute* (*Fine Arts and Design*) in the field of the basic theory of fine arts. *Journal of Beijing Dance Academy* discusses dance theory. *Chinese Music*, *Music Research*, *Musicology in China* studies music research theory. *Journal of College Chinese Traditional Opera* and *Theatre Arts* focuses on drama research. The magazine *Art and Design Research* targets fashion design. *Chinese Calligraphy* is designed for calligraphy research. *Film Art* can be cited for film research. *National Arts Bimonthly* and *Ethnic Art Studies* are frequently updated for national art research (Braby et al., 2020). These journals in different art fields were all founded in the 20th century, focusing on the history of Chinese aesthetics. Therefore, these journals can enhance college students’ aesthetic cognition and mental health. They are believed to carry forward Chinese traditional culture and improve college students’ national cognition and cultural acceptance (Smith-Keiling et al., 2020). Accordingly, this work studies the influence of art journal reading on college students’ national cognition and cultural acceptance.

The research on college students’ national awareness and cultural acceptance falls into the psychology field (Schwartz et al., 2021). National cognition and cultural acceptance is an individual’s feeling influenced by the group culture (Anders and Olmstead, 2019). Citizens’ cultural acceptance of their motherland forms the special national spiritual force among the diverse world culture (Shea et al., 2019). This work defines college students’ artistic cognition as their identity of traditional Chinese art and studies how art journal reading impacts it. National awareness is crucial in the research of national psychology. Cognition is an individual’s dynamic knowledge acquisition process. In particular, national cognition is an overall cognitive consistency of specific national groups (Gómez, 2019). It varies in lifestyles, values, and national characters of different nationalities worldwide (Stein et al., 2019), or the internalized national culture. College students’ cultural acceptance directly affects their national cognition.

### Research objectives

This work aims to explore the training mode of college students’ national cognition and cultural acceptance. Firstly, several hypotheses are proposed from the Educational Psychology perspective and deep learning (DL). Secondly, a questionnaire survey (QS) on college students’ national cognition and cultural acceptance is designed. The data on college students’ national cognition and cultural acceptance under the influence of art journal reading is collected. The collected data are analyzed by Statistical Product and Service Solutions (SPSS) 25.0. Finally, the art journal reading guidance system is constructed. Innovatively, this work concretizes national cognition and cultural acceptance into four indexes to facilitate measurement. The research results have a positive effect on enhancing college students’ national cognition and cultural acceptance and improving college students’ national self-confidence.

## Literature review

Mental health mainly studies people’s cognition. Cognitive activities are the basis and origin of all people’s psychological activities (Sabella, 2021). Cognition is a major intermediary process representing internal and external events and organizational forms. Also, cognition reflects an individual’s behavior and environment (Seow et al., 2021). Psychologists contend that cognition is an information processing process, a symbolic psychological operation, and a group of activities related to reasoning, perception, learning, judgment, and memory. Cognition is a part of psychological activities, the process of the brain perceiving the world through interaction with objective events involving rational thinking. People receive, process, and respond to information through cognitive processes. Furthermore, cognition includes basic cognitive activities, such as perception and feeling, and complex cognitive activities, such as imagination and thinking. Psychological activity is a reflection of objective reality.

Here, cognition is defined broadly, including individual cognitive activities, such as memory, feeling, thinking, and social cognition. Individual cognitive activities may differ greatly. The cognitive structure is also known as the archetype, which refers to an individual’s existing cognitive experience (Hartas, 2021). When an individual is faced with a problematic situation or stimulus, assimilation will be produced. That is, the existing cognitive structure is checked with the situation, the corresponding cognitive function is produced, and the new experience is incorporated into the existing experience structure. Assimilation can incorporate external information into the formed cognitive structure and use it in future new situations (Tokur and Kocoglu, 2021). The new situation is adapted into the cognitive structure, namely, the adaptation, which is another cognitive process by adjusting the original cognitive structure according to the new information and situation. Adaptation can improve intelligence periodically and reorganize cognition structure.

In terms of individual psychological structure, people’s response to external stimuli is always based on a certain psychological structure, namely, a specific experience. The analysis of the human brain’s advanced psychological activities shows that memory, feeling, thinking, and perception are basic human cognitive processes. People understand the objective world, selectively accept the environmental information, and respond to various stimuli through cognition. Besides, people accept and process external information by cognition. Human cognition includes the cognition of various objects in the natural environment and the cognition of various social phenomena, people, and society. People’s cognitive activities are based on previous experience and are the response to objective reality. In this case, groups and individuals with different living environments and life experiences have different cognitive styles. For example, due to geographical diversity and life experience, Chinese people may have different national cognition and cultural acceptance. People also show different psychology in national cognition and cultural acceptance. However, the core emotion brought by national cognition and cultural acceptance is the same: self-confidence. The designed learning guidance management system belongs to the learning system. The learning system can be divided into the Mobile learning (M-learning) system, personalized learning system, adaptive learning system, ubiquitous learning system, and collaborative learning system.

Internationally, the research on learning systems has seen a much earlier development. In the 1990s, European and American countries first researched M-learning, and foreign countries took M-learning as the future of online education (Peltz et al., 2021). At the beginning of the 21st century, the United States *Department of Education* planned and launched the mobile education scientific research project, established in the Human-Computer Interaction (HCI) research room of Kyushu University at Berkeley. The M-learning project led by European and American countries has solved the unequal distribution of national educational resources. The project uses a minimalist counting method to promote learning information and learning experience to a large number of learners. More importantly, it allows learners to participate in learning for life. Based on the M-learning project, British researchers have analyzed the learning characteristics of some teenagers in Europe. According to the learning behavior habits of these learners, they have developed an education site that facilitates teenagers’ mobile learning. To stimulate their interest in learning, researchers pay more attention to the fashionable and popular materials teenagers are interested in when selecting learning content. Domestically, research on M-learning projects has started relatively late. Peking University launched an M-learning project at the beginning of the 21st century. Domestic Internet giants have entered online education and changed the traditional education model (Parker et al., 2021). The research on personalized learning systems began in the 1970s. With increasing research on intelligent teaching systems, personalized customization systems have appeared as the core. At the beginning of the 21st century, American scholars first released the personalized learning system. China’s first personalized learning system was released in 2006. The research of personalized learning systems focuses on personalized service and user model construction. An adaptive learning system can generate adaptive learning content according to the characteristics of learners. The research on the adaptive system worldwide focuses on the overall theoretical modeling. The ubiquitous learning system is the application of education using ubiquitous computer technology. It creates a ubiquitous learning environment that allows learners to learn anytime and anywhere. The current research on ubiquitous learning systems focuses on ubiquitous technology. Lastly, the collaborative learning systems is mainly aimed at the collaborative learning ability based on networks, including online conference systems, online chat systems, learning tracking systems, group project organization systems, student self-evaluation systems, content search systems, and performance management system.

From the perspective of Educational Psychology and DL, the impact of art journal reading on college students’ national cognition and cultural acceptance is studied. Individuals may have different national cultural acceptance, but this difference is relatively small among a group of people in the same country. Generally, there should be no deviation in the overall direction of college students’ national culture cognition. There is a need for college students to take a positive view of Chinese national culture cognition. Positive national culture cognition will improve college students’ self-confidence. This work analyses the impact of art journal reading on college students’ national cognition and cultural acceptance and puts forward the journal reading guidance management system. The following sections will first put forward assumptions and design a QS on college students’ national cognition and cultural acceptance. Then, it analyses the QS results and builds a journal reading guidance system. Detailed research methods will be given in the next section.

## Research model

Educational Psychology can promote students’ autonomous learning. This work will be carried out based on Educational Psychology to positively influence students’ thinking and promote national cognition and cultural acceptance. Based on Educational Psychology, art journals’ influence on college students’ national cognition and cultural acceptance will be studied in the following.

### Research hypothesis

Through DL, learners independently learn, explore, understand, and construct national identity and feedback on the results after reading journals. The research hypothesis and QS design also refer to the concept of DL.

The current research on college students’ national cognition and cultural acceptance focuses on the identity crisis of how individuals identify themselves in a specific cultural atmosphere. With globalization, Western culture increasingly impacts China (Gattamorta et al., 2019), and the national cognition and cultural acceptance crisis is becoming ever-more serious among college students (Edele et al., 2020). In this social and cultural context, it is vital to cultivate college students’ national cultural self-confidence. To this end, reading art journals can create a reasonable and healthy cultural acceptance environment (Barnes et al., 2020). The influence of art journal reading on college students’ cultural acceptance is mainly reflected in the following aspects. First, by reading excellent Chinese traditional art and cultural journals, college students can master the core of Chinese traditional culture (Backhaus et al., 2020). Second, by reading the excellent revolutionary culture and artworks in journals, college students can understand the Red Spirit of the Communist Party of China (CPC) (Sajnani et al., 2020). Third, by reading China’s advanced socialist cultural works in art journals, college students can understand the socialist culture with Chinese characteristics (Krasovska et al., 2020).

The current situation of college students’ national cognition and cultural acceptance is mainly reflected in their understanding of national culture. Fifty-six nationalities in China have a different understanding of national culture. Generally speaking, Chinese college students face a crisis of national cognition and cultural acceptance (Lattie et al., 2019). With the popularity of the Internet and global cultural and economic exchanges, the materialism-oriented Western culture has attracted many young people. This threatens the development and inheritance of Chinese national culture (Liu et al., 2019). In this case, the essence of art journals can help college students enhance their national cognition and cultural acceptance. Additionally, art journals involve Chinese traditional culture, national art, and national art research. They play an essential role in cultivating college students’ national cognition and cultural acceptance.

College students’ national cognition and cultural acceptance is analyzed collectively and comprehensively. Then, the impact of art journal reading is examined by considering college students’ understanding of Chinese traditional culture, excellent revolutionary culture, advanced socialist culture, and Chinese national identity cognition. Finally, it summarizes the impact of art journal reading on college students’ national cognition and cultural acceptance (Lipson et al., 2019).

Following the literature review, the following hypotheses are put forward, integrating previous studies and making a breakthrough in the basic situation of college students.

Hypothesis 1: Art journal reading has a significant impact on the national cognition and cultural acceptance of college students of different genders.

Hypothesis 2: Art journal reading has a significant impact on the national cognition and cultural acceptance of college students of different nationalities.

Hypothesis 3: Art journal reading has a significant impact on the national cognition and cultural acceptance of college students in different disciplines.

Hypothesis 4: Under the influence of art journal reading, college students with good family backgrounds have greatly improved their national cognition and cultural acceptance.

Hypothesis 5: Art journal reading has a significant impact on the national cognition and cultural acceptance of college students with different political statuses.

Hypothesis 6: The number of art journals read by college students positively correlates with their national cognition and cultural acceptance.

### Deep learning technology and method

Based on the previous content, a journal reading guidance system is built to improve college students’ national cognition and cultural acceptance. Several machine learning (ML) algorithms, such as computer vision and deep reinforcement learning (DRL), are employed.

The visual geometry group (VGG) is an effective deep network framework chosen to identify students’ evaluations. DRL requires frequent interaction between agents and the environment. Specifically, when an agent completes an action, it gets a certain reward. To get an optimal action, the agent must consider the long-term benefits of multiple action sequences after the action to maximize the future reward. Therefore, the intelligent agent keeps trying, interacts with the environment, and gradually improves its own strategy. The problem of intelligent guidance belongs to some observable reinforcement learning problems. In some reinforcement learning models, agents can fully observe the current environmental state. This is not in line with the scenario of the proposed intelligent reading guidance system. Students’ learning is adjusted through the observation of students’ learning results. In other words, the proposed intelligent guidance system here is a partially observable reinforcement learning problem.

The development platform of the proposed learning guidance system includes an open-source training system of MIT, the ML algorithm library Scikit-learn, the distribution version of Python: Anaconda integrating multiple scientific computing packages, and the reinforcement learning algorithm library Rllab, and the ML framework TensorFlow.

### Construction of journal reading guidance system

**FIGURE 1:**
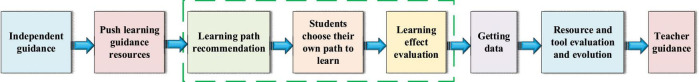
Journal reading guidance system.

Based on the previous content, this section constructs a journal reading guidance system for college students’ national cognition and cultural acceptance, as shown in Figure 1.

Figure 1 shows the designed journal reading guidance system. Obviously, the system is composed of eight parts: independent guidance, resources recommendation, learning path recommendation, students’ self-selected path learning, learning effect evaluation, data acquisition, resource and tool evaluation and evolution, and teacher guidance. Of these, learning path recommendation, students’ self-selected path learning, and learning effect evaluation are the core links. The designed journal reading guidance system is completely independent. Ideological and political teachers upload journal resources to the resource database, which is recommended by the system according to the push strategy or independently selected by students. Teachers provide students with journal resources that can support personalized exploration. According to the specific subjects, household registration, political status, and reading volume, different paths of guidance resources are formed to achieve personalized and classified teaching. Students can choose tailor-made learning paths and feed the journal reading results back. The system uses the feedback to recommend the journal resources accurately and improve students’ national cognition and cultural acceptance. The MIT open-source practice system provides the front end and database of the system. Heuristic and recommendation algorithms based on DRL are used for the journal recommendation.

Here, the core of the learning guidance system: the recommendation module, is mainly introduced. The selected open-source exercise system is a learning guidance system with browser-server architecture, which interacts with users through web browsing. The browsing results of students need to be checked before recommendation. Questions will be set at the end of each chapter of the journal. The check module scores the student-submitted reports and stores the scores in the log and context data structure for the recommendation module. Then, the system will recommend the next journal based on the report evaluation. The recommendation module evaluates the students’ historical information before pushing another art journal. In this part, tutor.py is modified, and the handle_recommend function is added, which calls the evaluation function based on the latest student reading records to calculate and recommend the following journal. The following paper will design a QS to compare students’ national identity differences before and after using the proposed learning guidance system.

## Experimental design and performance evaluation

### Experimental materials

A QS is designed through prediction and expert evaluation based on domestic and international research theories. The QS includes the basic situation of college students and their understanding of Chinese traditional culture, Chinese excellent revolutionary culture, and Chinese advanced socialist culture before and after reading the art journal.

Here, 200 students, with 50 from each of the four grades, are selected. The specific division is shown in Table 1.

**TABLE 1:** The basic items of the QS.

Division dimension	Condition	Number/person
Gender	Female	80
	Male	120
Ethnicity	Han	142
	Minority	58
Discipline	Literature and history	90
	Science and engineering	110
Family background	Urban residence registration	116
	Rural household registration	84
Political status	Party member	47
	League member	153
Art journals read	Less than five copies	98
	5–10 copies	87
	More than 10 copies	15

Table 1 presents that the basic items of the QS include gender, ethnicity, discipline, family background, political status, and art journals read. With regard to nationalities, respondents are divided into Han and Minorities.

### Experimental environment

Here, Southeast University is selected as the study area. For more significant research results, non-art major students from the regular university who seldom read art journals are recruited. Based on the importance of online learning in teaching proposed by Shen et al. (2019), art journals are provided online for students to read (Shen et al., 2019). Specifically, two rounds of QS with the same content are issued, lasting 1 month. The first round of the experiment does not use, and the second round uses the journal reading guidance system. All grades are covered to ensure the representativeness of the QS results. Altogether, 220 QSs are distributed, with 200 returned. All the returned QSs are valid. The distribution of detailed QS results is shown in Table 1.

### Parameters setting

The QS (as Appendix) includes the basic information of college students, and the specific questions are set from four aspects: Chinese traditional culture, Chinese excellent revolutionary culture, Chinese advanced socialist culture, and Chinese national identity cognition, as shown in Table 2.

**TABLE 2:** Question setting of the QS.

Main aspects	Specific questions
Chinese traditional culture	Can you answer some questions about Chinese traditional culture that you know and briefly introduce them? (if the answer involves more than three traditional cultures, it means “know some.”)
	Do you think you love Chinese traditional culture?
	Do you think you are willing to inherit Chinese traditional culture?
Chinese excellent revolutionary culture	Do you think you understand Chinese excellent revolutionary culture?
	Do you think you love Chinese excellent revolutionary culture?
	Do you think learning about excellent revolutionary culture is helpful to self-construction?
Chinese advanced socialist culture	Do you think you understand the Chinese advanced socialist culture?
	Do you think Chinese advanced socialist culture is superior to Western capitalist culture?
	Do you think you are willing to participate in the construction of Chinese advanced socialist culture?
Chinese national identity cognition	Do you think you know the Chinese nation? You can give a brief answer about your understanding of the Chinese nation (A brief answer to the total number of nationalities and traditional culture of the Chinese nation means “know some”)
	Do you recognize your identity as a Chinese nation?
	Do you think the Chinese nation is the best in the world?

Table 2 shows that the QS is designed with 12 specific indicators to explore the current situation of college students’ national cognition and cultural acceptance.

The QS data are processed with SPSS25.0, and the subjective questions are calculated manually (Melcher et al., 2020). The indices of analytical results include the mean, *T* (Independent variable test value), *P*-value (test result), *F* (variance test), and Sig. (significance). *P*-value > 0.05 indicates no significant data difference. 0.05 ≥ *P*-value ≥ 0.01 indicates a significant data difference, and *P*-value < 0.01 indicate an extreme significant data difference (Chow and Choi, 2019).

### Performance evaluation

#### Analysis of the influence of gender on college students’ national cognition and cultural acceptance

The data results of the national cognition and cultural acceptance of college students of different genders before and after reading art journals are shown in Table 3.

**TABLE 3:** Differences in the national cognition and cultural acceptance of college students of different genders.

	Aspects	Gender	Mean	*T*	*P*-value	*F*	Sig.
Before art journal reading	Chinese traditional culture	Female	4.31	1.526	0.060	2.783	0.051
		Male	4.23				
	Chinese excellent revolutionary culture	Female	4.42	2.635	0.070	3.710	0.045
		Male	4.39				
	Chinese advanced socialist culture	Female	4.32	−1.639	0.058	4.929	0.040
		Male	4.33				
	Chinese national identity cognition	Female	4.30	3.742	0.056	5.731	0.040
		Male	4.27				
	Overall cognition	Female	4.33	−0.562	0.671	0.7892	0.829
		Male	4.31				
After art journal reading	Chinese traditional culture	Female	5.08	1.652	0.052	2.891	0.068
		Male	4.99				
	Chinese excellent revolutionary culture	Female	4.99	2.782	0.051	2.989	0.049
		Male	4.89				
	Chinese advanced socialist culture	Female	4.90	−2.672	0.055	4.012	0.061
		Male	4.97				
	Chinese national identity cognition	Female	4.98	2.869	0.056	5.829	0.011
		Male	4.90				
	Overall cognition	Female	4.99	−0.566	0.728	0.8017	0.821
		Male	4.94				

Table 3 shows that before and after reading art journals, girls’ national cognition and cultural acceptance is higher than boys. Especially, their cognition of Chinese traditional culture, excellent revolutionary culture, and Chinese national identity are higher than boys. By comparison, male students have a higher cognition of Chinese advanced socialist culture than female students. When *P*-value is greater than 0.05, there is no significant difference between male and female students.

**FIGURE 2:**
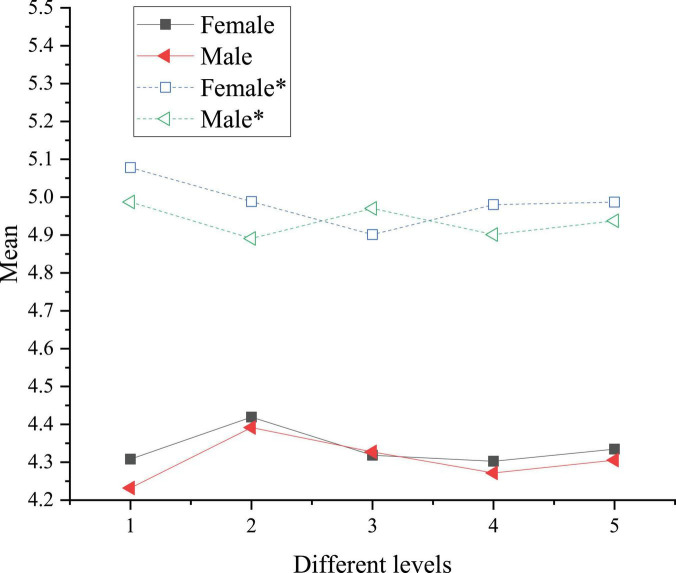
Comparison of the national cognition and cultural acceptance of college students of different genders before and after reading art journals. * denotes post-test sample.

A comparison chart is plotted based on the means in Table 3, as shown in Figure 2.

In Figure 2, numbers 1, 2, 3, 4, and 5 represent Chinese traditional culture, excellent revolutionary culture, advanced socialist culture, Chinese national identity cognition, and overall cognition, respectively. The asterisk curve shows the cognition after art journal reading. The result shows that art journals can enhance college students’ national cognition and cultural acceptance. Female college students show the highest promotion rate in Chinese traditional culture, reaching 16.3%. Women are more emotional and empathic than men. Thus, they have a stronger national emotion in Chinese traditional culture, excellent revolutionary culture, and Chinese national identity cognition. This conclusion is consistent with the relationship between empathy and competitiveness proposed by Rogoza et al. (2018), namely, the positive role of high empathy in emotional learning (Rogoza et al., 2018). By comparison, men have a stronger objective analysis (Ma et al., 2020), so their cognitive level of Chinese advanced socialist culture is higher than that of women.

#### Analysis of the results of the national cognition and cultural acceptance of college students of different nationalities

Table 4 displays the national cognition and cultural acceptance of Han and Minority college students before and after art journal reading.

**TABLE 4:** Comparison of the national cognition and cultural acceptance of college students of different nationalities.

	Aspects	Ethnicity	Mean	*T*	*P*-value	*F*	Sig.
Before art journal reading	Chinese traditional culture	Han	4.30	−1.506	0.003	4.723	0.051
		Minority	4.34				
	Chinese excellent revolutionary culture	Han	4.42	−2.645	0.034	5.770	0.046
		Minority	4.45				
	Chinese advanced socialist culture	Han	4.31	−1.536	0.041	9.929	0.044
		Minority	4.32				
	Chinese national identity cognition	Han	4.33	−3.895	0.002	10.731	0.041
		Minority	4.38				
After art journal reading	Chinese traditional culture	Han	4.80	−1.658	0.001	4.891	0.048
		Minority	4.88				
	Chinese excellent revolutionary culture	Han	4.89	−2.784	0.032	5.989	0.049
		Minority	4.89				
	Chinese advanced revolutionary culture	Han	4.90	−2.293	0.045	9.015	0.041
		Minority	4.97				
	Chinese national identity cognition	Han	4.92	−2.563	0.002	10.829	0.011
		Minority	4.98				

Table 4 implies that before and after reading art journals, the overall level of the national cognition and cultural acceptance of Minority students is higher than that of Han students, with a *P* < 0.05. Specifically, the mean scores of the Minority students are higher than Han students in Chinese traditional culture, excellent revolutionary culture, advanced socialist culture, and Chinese national identity cognition.

**FIGURE 3:**
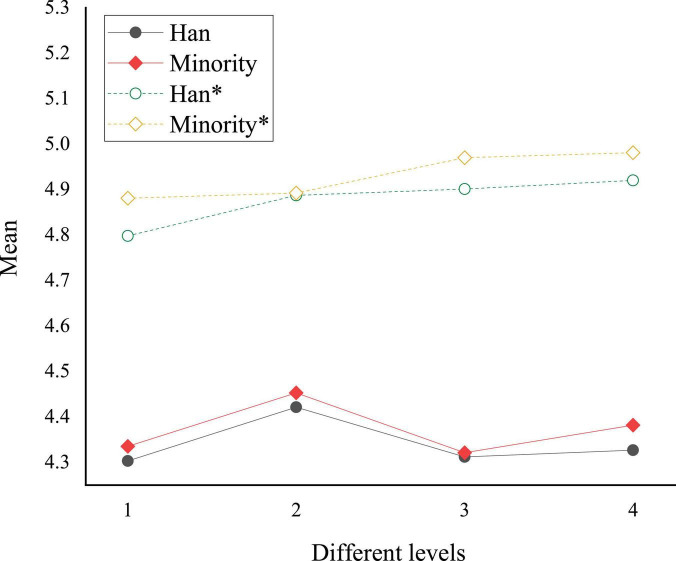
Comparison of the national cognition and cultural acceptance of college students of different nationalities before and after reading art journals. * denotes post-test sample.

A comparison chart is plotted based on the means in Table 4, as shown in Figure 3.

In Figure 3, numbers 1, 2, 3, and 4 represent Chinese traditional culture, excellent revolutionary culture, advanced socialist culture, and Chinese national identity cognition, respectively. The asterisk curve indicates cognition after reading art journals. Obviously, art journal reading improves college students’ cultural acceptance. Minority students’ national cognition and cultural acceptance level before and after reading art journals is higher than Han students. Minority college students have the highest cognitive improvement rate in socialist advanced cultures, reaching 15.1%. Similarly, Han college students have the highest cognitive improvement rate in advanced socialist culture, about 13.7%. Probably, this is because college students have less knowledge of advanced socialist culture in peacetime, and the theoretical knowledge in books can quickly supplement this part of knowledge. Meanwhile, Minorities might have stronger beliefs than Han people, so the overall cognition of Minority college students is higher than that of Han students (Barnett et al., 2019). Minorities have their own national culture and tradition (Li et al., 2021), so they have a strong national will. Minorities love their ethnic identity and the Chinese nation.

#### Comparative analysis of the national cognition and cultural acceptance of college students in different majors

Table 5 indicates the national cognition and cultural acceptance data of college students in Literature and History and Science and Engineering before and after reading art journals.

**TABLE 5:** Comparison of the national cognition and cultural acceptance of college students in different majors.

	Aspects	Discipline	Mean	*T*	*P*-value	*F*	Sig.
Before art journal reading	Chinese traditional culture	Literature and History	4.34	0.506	0.763	5.723	0.021
		Science and Engineering	4.34				
	Chinese excellent revolutionary culture	Literature and History	4.42	−2.645	0.046	6.780	0.036
		Science and Engineering	4.36				
	Chinese advanced socialist culture	Literature and History	4.31	−1.536	0.331	8.929	0.014
		Science and Engineering	4.33				
	Chinese national identity cognition	Literature and History	4.33	2.825	0.060	6.731	0.009
		Science and Engineering	4.33				
After art journal reading	Chinese traditional culture	Literature and history	4.90	0.658	0.810	4.851	0.028
		Science and Engineering	4.88				
	Chinese excellent revolutionary culture	Literature and History	4.88	−2.714	0.032	8.909	0.019
		Science and Engineering	4.87				
	Chinese advanced socialist culture	Literature and History	4.80	−1.993	0.085	6.010	0.051
		Science and Engineering	4.90				
	Chinese national identity cognition	Literature and History	4.92	2.363	0.064	7.829	0.021
		Science and Engineering	4.93				

Table 5 displays that Literature and History and Science and Engineering students have a high level of national cognition and cultural acceptance. The overall cognition of Literature and History students is higher than those of Science and Engineering students in Chinese traditional culture and excellent revolutionary culture and lower in Chinese socialist advanced culture and Chinese national identity cognition. The *P*-value of Chinese excellent revolutionary culture is less than 0.05, showing that the difference in Chinese excellent revolutionary culture is significant in college students from different disciples.

**FIGURE 4:**
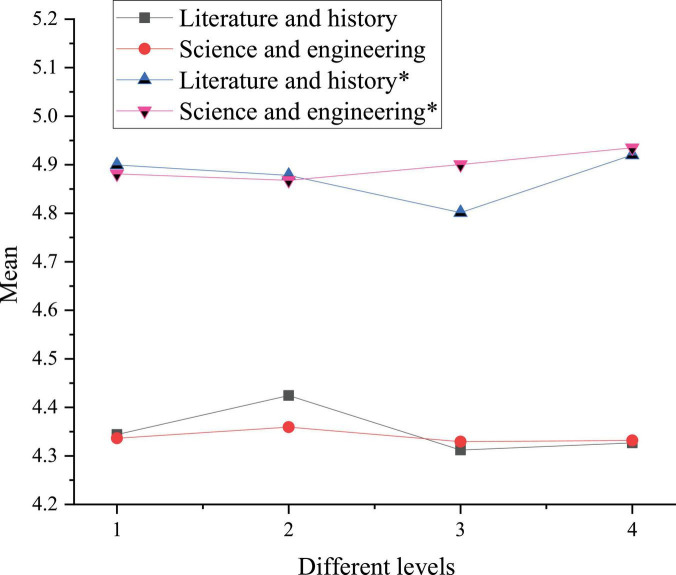
Comparison of the national cognition and cultural acceptance between college students of different disciplines before and after reading art journals. * denotes post-test sample.

A comparison chart is plotted based on the means in Table 6, as shown in Figure 4.

**TABLE 6:** Comparison of the national cognition and cultural acceptance of college students from different family backgrounds.

	Aspects	Family background	Mean	*T*	*P*-value	*F*	Sig.
Before art journal reading	Chinese traditional culture	Town registered permanent residence	4.31	−1.864	0.163	0.523	0.459
		Registered rural residence	4.30				
	Chinese excellent revolutionary culture	Town registered permanent residence	4.42	0.645	0.046	0.780	0.736
		Registered rural residence	4.36				
	Chinese advanced socialist culture	Town registered permanent residence	4.31	−5.536	0.231	0.929	0.814
		Registered rural residence	4.30				
	Chinese national identity cognition	Town registered permanent residence	4.33	−3.825	0.350	0.734	0.909
		Registered rural residence	4.33				
After art journal reading	Chinese excellent revolutionary culture	Town registered permanent residence	4.89	−2.658	0.200	0.851	0.528
		Registered rural residence	4.86				
	Chinese excellent revolutionary culture	Town registered permanent residence	4.87	1.137	0.032	0.902	0.619
		Registered rural residence	4.81				
	Chinese advanced socialist culture	Town registered permanent residence	4.82	−5.224	0.435	0.015	0.851
		Registered rural residence	4.81				
	Chinese national identity cognition	Town registered permanent residence	4.91	−2.917	0.320	0.829	0.821
		Registered rural residence	4.93				

In Figure 4, numbers 1, 2, 3, and 4 stand for Chinese traditional culture, excellent revolutionary culture, advanced socialist culture, and Chinese national identity cognition, respectively. The asterisk curve means cognition after reading art journals. Apparently, art journal reading has greatly improved the cognitive level of Science and Engineering students. The cognitive level of Chinese national identity is the highest, with an improvement rate of 13.8%, and that of Han students is 13.6%. The reason may be that Science and Engineering students usually have less contact with art journals, and their major curriculums seldom involve excellent revolutionary culture (Chang et al., 2019). Therefore, after reading art journals, the overall level has improved dramatically.

#### Comparative analysis of the national cognition and cultural acceptance of college students from different family backgrounds

Table 6 reveals the data on the national cognition and cultural acceptance of urban and rural college students before and after reading art journals.

In Table 6, urban college students’ cognition is higher in Chinese traditional culture, excellent revolutionary culture, and advanced socialist culture and lower in the Chinese national identity than rural students. The overall cognitive of urban college students is higher than that of rural college students. Because *P*-value is less than 0.05, the difference in Chinese excellent revolutionary culture is significant between rural and urban college students.

**FIGURE 5:**
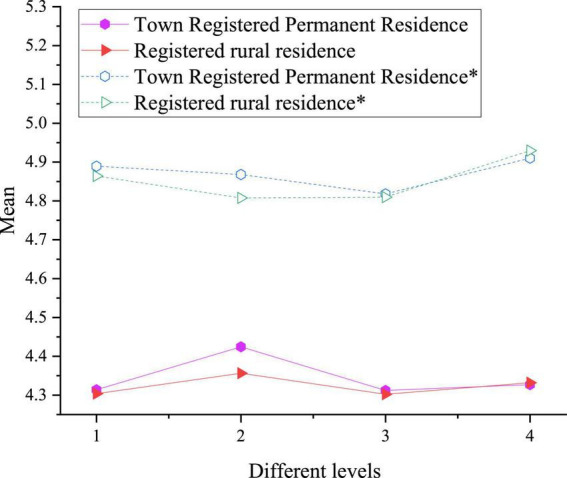
Comparison of the national cognition and cultural acceptance of college students from different family backgrounds before and after reading art journals. * denotes post-test sample.

A comparison chart is plotted based on Table 7, as shown in Figure 5.

**TABLE 7:** Comparison of the national cognition and cultural acceptance of college students with the different political status.

	Aspects	Political status	Mean	*T*	*P*-value	*F*	Sig.
Before art journal reading	Chinese traditional culture	Party member	4.51	−3.864	0.003	3.523	0.0419
		League member	4.40				
	Chinese excellent revolutionary culture	Party member	4.52	0.640	0.045	3.780	0.0726
		League member	4.46				
	Chinese advanced socialist culture	Party member	4.41	−0.536	0.031	5.929	0.0819
		League member	4.30				
	Chinese national identity cognition	Party member	4.43	−0.225	0.050	3.734	0.0909
		League member	4.40				
After art journal reading	Chinese traditional culture	Party member	4.98	−2.958	0.001	3.851	0.0525
		League member	4.96				
	Chinese excellent revolutionary culture	Party member	4.97	0.137	0.032	3.902	0.0614
		League member	4.89				
	Chinese advanced socialist culture	Party member	4.92	−0.224	0.035	5.015	0.0891
		League member	4.81				
	Chinese national identity cognition	Party member	4.90	−0.417	0.020	3.829	0.0820
		League member	4.87				

In Figure 5, numbers 1, 2, 3, and 5 denote Chinese traditional culture, excellent revolutionary culture, advanced socialist culture, and Chinese national identity cognition, respectively. The asterisk curve is cognition after reading art journals. Evidently, before and after reading art journals, the national cognition and cultural acceptance curves of students from different family backgrounds are consistent. After reading art journals, the Chinese national identity of students from registered rural residents has improved dramatically, about 13.2%. The reason is that the family background directly affects the cultural acceptance of students (Jao et al., 2019). Compared with students from town registered permanent residents, rural residents receive less aesthetic education and are hardly exposed to the artistic atmosphere during school education. Therefore, by reading art journals, the national cognition and cultural acceptance of students from registered rural residents will be greatly improved.

#### Analysis of the results of the national cognition and cultural acceptance of college students with different political statuses

Table 7 shows the national cognition and cultural acceptance data of college student Party members and League members before and after reading art journals.

In Table 7, the *P*-values are less than 0.05, so there are significant differences in national cognition and cultural acceptance among college students with different political statuses. The overall national cognition and cultural acceptance level of Party member college students is higher than that of League member college students, especially in Chinese advanced socialist culture.

**FIGURE 6:**
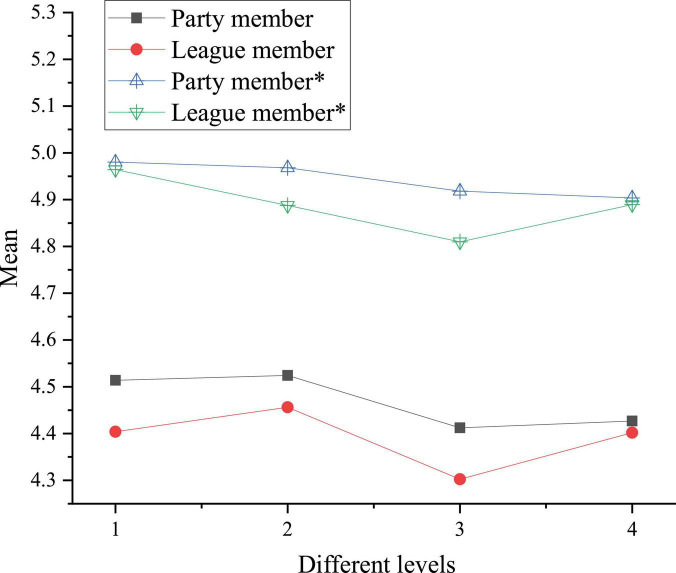
Comparison of the national cognition and cultural acceptance of college students with different political statuses before and after reading art journals. * denotes post-test sample.

A comparison chart is plotted based on Table 7, as shown in Figure 6.

In Figure 6, numbers 1, 2, 3, and 4 represent Chinese traditional culture, excellent revolutionary culture, advanced socialist culture, and Chinese national identity cognition, respectively. The asterisk curve shows cognition after reading art journals. Figure 6 suggests that the League members have the highest promotion rate in Chinese traditional culture cognition, which is 12.7%. Reading art journals has strengthened the cognition of the League members in Chinese traditional culture and Chinese national identity, and the level after reading is close to that of the Party members. The level of Party members is higher than that of League members in all aspects. The reason may be that the Communist Party is more mature than the Communist Youth League. Besides, Party’s construction is stricter than League’s, and Party pays more attention to the ideological construction of Party members in ordinary times. Therefore, the national cognition and cultural acceptance of Party members is higher than that of League members.

#### Analysis of the influence of different reading volumes of art journals on college students’ national cognition and cultural acceptance

The respondents have read 0–1 copies of art journals by default. The QS is issued after the respondents read five copies, 5–10 copies, and more than ten copies of art journals. The data analysis is shown in Table 8.

**TABLE 8:** Comparison of the national cognition and cultural acceptance of college students after reading different numbers of art journals.

	Aspects	Reading quantities	Mean	*T*	*P*-value	*F*	Sig.
Before art journal reading	Chinese traditional culture	0–1 copies	4.33	−2.864	0.063	2.523	0.0419
	Chinese excellent revolutionary culture		4.32	0.445	0.145	1.880	0.0226
	Chinese advanced socialist culture		4.30	−0.436	0.063	2.189	0.0219
	Chinese national identity cognition		4.33	−0.569	0.059	1.739	0.0919
After art journal reading	Chinese traditional culture	<5 copies	4.48	−2.858	0.034	1.622	0.125
		5–10 copies	4.66				
		>10 copies	4.98				
	Chinese excellent revolutionary culture	<5 copies	4.57	0.537	0.033	2.902	0.214
		5–10 copies	4.89				
		>10 copies	5.09				
	Chinese advanced socialist culture	<5 copies	4.52	−0.328	0.012	3.015	0.211
		5–10 copies	4.71				
		>10 copies	4.89				
	Chinese national identity cognition	<5 copies	4.60	−0.483	0.041	1.829	0.720
		5–10 copies	4.89				
		>10 copies	4.99				

Table 8 illustrates that before reading art journals, college students have no significant difference in national cognition and cultural acceptance. However, after art journal reading, there are significant differences in college students’ national cognition and cultural acceptance. Specifically, the differences in Chinese traditional culture, excellent revolutionary culture, advanced socialist culture, and Chinese national identity cognition have become more significant.

**FIGURE 7:**
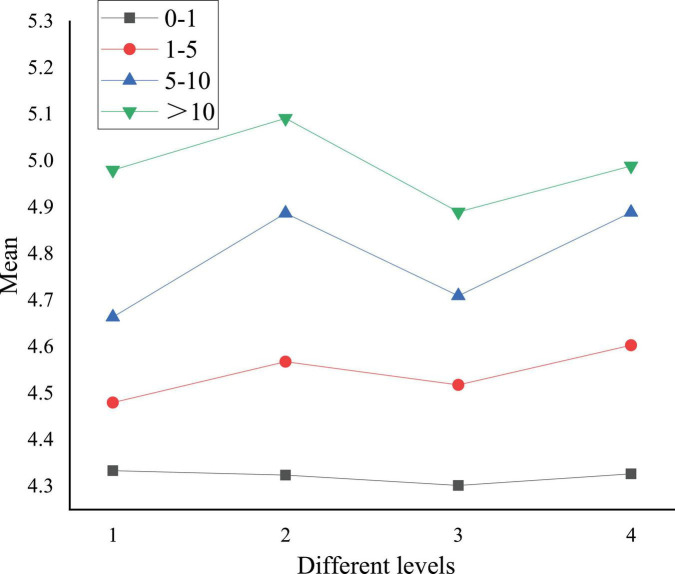
The influence of reading volume of art journals on college students’ national cognition and cultural acceptance.

A comparison chart is plotted using means in Table 8, as shown in Figure 7.

In Figure 7, numbers 1, 2, 3, and 4 refer to Chinese traditional culture, excellent revolutionary culture, advanced socialist culture, and Chinese national identity cognition, respectively. The asterisk curve means the cognition after art journal reading. Figure 7 implies that reading more art journals improves college students’ national cognition and cultural acceptance. In particular, the excellent revolutionary culture and the Chinese national identity cognition are greatly improved. The improvement of Chinese traditional culture and advanced socialist culture is slight. The promotion rate of cognition on excellent revolutionary culture is the highest after students read more than ten art journals, which is 17.8%. All dimensions increase with the increase of the number of art journals read.

## Discussion

Based on the above analysis, six hypotheses can be verified. Art journal reading has the same impact on the national cognition and cultural acceptance of college students of different genders; thus, Hypothesis 1 holds. Minority students have a higher national cognition and cultural acceptance than Han students. Reading art journals play a positive role in improving them; hence, Hypothesis 2 is not tenable. The improvement of Science and Engineering students after reading art journals differs from that of Literature and History students; thus, Hypothesis 3 holds. The national cognition and cultural acceptance of students from rural and town registered permanent residences have improved after reading art journals; hence, Hypothesis 4 holds. After art journal reading, the promotion of the national cognition and cultural acceptance of members of the Communist Youth League is higher than that of Party members; thus, Hypothesis 5 holds. Finally, the greater the volume of reading art journals is, the higher the level of college students’ national cognition and cultural acceptance is; hence, Hypothesis 6 holds.

## Conclusion

### Research contribution

Based on educational psychology, the research topic is deeply analyzed following the literature review and QS design. The results manifest that reading art journals can improve college students’ national cognition and cultural acceptance. The impact of reading art journals on the national cognition and cultural acceptance of college students of different genders and ethnic groups is the same. However, the impact on college students with different disciplines, family backgrounds, and political affiliations differs. The more students read art journals, the higher their national cognition and cultural acceptance is. The results can provide a reference for promoting college students’ national cognition and cultural acceptance in the new era.

### Future works and research limitations

There are also some shortcomings: the selection of evaluation indicators for national cognition and cultural acceptance is insufficient, and indicators’ representativeness needs further improvement. The constructed journal reading guidance system has only a framework and has not been refined. Therefore, more indicators will be selected in the follow-up to obtain more convincing and feasible research results. In the future, the journal reading guidance system will be deepened, and the national cognition and cultural acceptance of college students will be studied in the context of HCI.

## Data availability statement

The raw data supporting the conclusions of this article will be made available by the authors, without undue reservation.

## Ethics statement

The studies involving human participants were reviewed and approved by Southeast University Ethics Committee. The patients/participants provided their written informed consent to participate in this study. Written informed consent was obtained from the individual(s) for the publication of any potentially identifiable images or data included in this article.

## Author contributions

All authors listed have made a substantial, direct, and intellectual contribution to the work, and approved it for publication.

## Appendix

### Questionnaire on college students’ national cognition and cultural acceptance

Dear students:

Thank you for taking the time to assist us in this survey. Here, we express our heartfelt thanks to you! Please choose the most suitable one after each question according to your situation and first feeling. There is no right or wrong choice, which only shows your thoughts. The questionnaire is anonymous, and the results of each student’s answer will be kept strictly confidential. Please treat each question carefully and do not miss it. Thank you for your cooperation.

#### Part 1 (for questions 1–6, please circle or tick the option number of each question or provide your own answer)

(1) Gender (A) Male (B) Female

(2) Ethnic group (A) Han (B) Minority

(3) Political status (A) League member (B) Party members (including probationary Party members) (C) The mass

(4) Major (A) Literature and History (B) Science and Engineering (C) Arts

(5) Household registration (A) Urban (B) Town (C) Village

(6) The number of art journals you usually read monthly (A) Less than five copies (B) 5∼10 copies (C) Over ten copies

#### Part 2 (for questions 1–12, please circle or tick the most appropriate option number)

##### Chinese traditional culture

(1) Can you answer some questions about Chinese traditional culture that you know and briefly introduce them? (more than three indicates “you know some”)

(2) Do you think you like Chinese traditional culture?

(A) Absolutely not (B) No (C) General (D) Yes (E) Very much

(3) Do you think you are willing to inherit Chinese traditional culture?

(A) Absolutely not (B) No (C) General (D) Yes (E) Very much

##### Chinese excellent revolutionary culture

(4) Do you think you know the excellent revolutionary culture of China?

(A) Absolutely not (B) No (C) General (D) Yes (E) Very much

(5) Do you think you like Chinese excellent revolutionary culture?

(A) Absolutely not (B) No (C) General (D) Yes (E) Very much

(6) Do you think learning about excellent revolutionary culture is conducive to self-construction?

(A) Absolutely not (B) No (C) General (D) Yes (E) Very much

##### Chinese advanced socialist culture

(7) Do you think you understand China’s advanced socialist culture?

(A) Absolutely not (B) No (C) General (D) Yes (E) Very much

(8) Do you think Chinese advanced socialist culture is superior to Western capitalist culture?

(A) Absolutely not (B) No (C) General (D) Yes (E) Very much

(9) Do you think you are willing to participate in the construction of Chinese advanced socialist culture?

(A) Absolutely not (B) No (C) General (D) Yes (E) Very much

##### Chinese national identity cognition

(10) Do you think you know the Chinese nation? You can briefly answer your understanding of the Chinese nation (a simple answer to the total number of nationalities and traditional cultures of the Chinese nation means “you have some understanding”).

(11) Do you admit that you are Chinese?

(A) Absolutely not (B) No (C) General (D) Yes (E) Very much

(12) Do you think the Chinese nation is the best nation in the world?

(A) Absolutely not (B) No (C) General (D) Yes (E) Very much
